# EVIDENCE OF THE ASSOCIATION BETWEEN SLEEP DURATION AND BLOOD PRESSURE IN ADOLESCENTS: A SYSTEMATIC REVIEW

**DOI:** 10.1590/1984-0462/2021/39/2019225

**Published:** 2020-08-05

**Authors:** Emanuela de Souza Gomes dos Santos, Orivaldo Florencio de Souza

**Affiliations:** a Instituto Federal de Educação, Ciência e Tecnologia do Acre, Rio Branco, AC, Brazil.; b Universidade Federal do Acre, Rio Branco, AC, Brazil.

**Keywords:** Adolescent, Arterial pressure, Sleep, Adolescente, Pressão arterial, Sono

## Abstract

**Objective:**

To review the epidemiological evidence of the association between sleep duration and blood pressure in adolescents.

**Data sources:**

We performed a systematic review of observational studies in Medline, Scopus, Lilacs, Web of Science, Science Direct databases and Virtual Libraries in English, Spanish and Portuguese published until September 2018. Studies were selected first by title and abstract, then by complete reading, according to the eligibility criteria. The reference list of selected articles was evaluated in order to retrieve relevant studies.

**Data synthesis:**

Initially, 1,455 articles were retrieved. After exclusion due to duplicity or not meeting the eligibility criteria, 13 articles were included in the review. Studies varied greatly in sample size (143 to 6,940 patients), methods of measuring blood pressure and sleep duration, cutoff points, categorization and adjustment of variables. The main evidence from the studies is that short sleep duration is associated with high blood pressure in adolescence, although the presence of association between high blood pressure and long sleep duration is possible, but not clear in the literature.

**Conclusions:**

Sleep duration, especially short duration, is associated with high blood pressure in adolescents. Such evidence draws attention to implications on cardiovascular health in this age group.

## RESUMO

**Objetivo:**

Discutir as principais evidências epidemiológicas da associação entre duração do sono e pressão arterial em adolescentes relatadas na literatura científica.

**Fonte de dados:**

Foi realizada uma revisão sistemática de estudos observacionais nas bases de dados do Sistema Online de Busca e Análise de Literatura Médica (MEDLINE), Scopus, Literatura Latino-Americana e do Caribe em Ciências da Saúde (Lilacs), Web of Science, ScienceDirect e bibliotecas virtuais nos idiomas inglês, espanhol e português para todo o período anterior a setembro de 2018. Foram selecionados trabalhos primeiramente pelo título e pelo resumo, em seguida pela leitura completa, conforme os critérios de elegibilidade. A lista de referência dos artigos selecionados foi avaliada a fim de recuperar estudos relevantes.

**Síntese dos dados:**

Inicialmente, foram recuperados 1.455 artigos. Após exclusões por duplicidade ou por não se enquadrarem nos critérios de elegibilidade, resultaram 13 artigos, que foram incluídos na revisão. Os estudos variaram bastante em tamanho de amostra (143 a 6.940), métodos de mensuração da pressão arterial e duração do sono, pontos de corte, categorização e ajuste de variáveis. As principais evidências trazidas pelos estudos são de que a curta duração do sono está associada à pressão arterial elevada na adolescência, embora não se descarte a possibilidade da relação entre pressão arterial elevada e longa duração do sono, que ainda não está clara na literatura.

**Conclusões:**

A duração do sono, principalmente a curta duração, está associada à pressão arterial elevada em adolescentes. Tais evidências chamam atenção para implicações sobre a saúde cardiovascular nessa faixa etária.

## INTRODUCTION

High blood pressure, which has been identified as a major public health epidemic,^1^ ^,^ ^2^ is an important risk factor for cardiovascular disease and is increasingly evident in children and adolescents.^3^ ^,^ ^4^ ^,^ ^5^ The results of the Study of Cardiovascular Risks in Adolescents (*Estudo dos Riscos Cardiovasculares em Adolescentes* - ERICA), conducted in Brazil, showed that 24% of adolescents have high blood pressure (prehypertension or hypertension) and 10% were classified as hypertensive.^5^ In addition, high blood pressure in adolescence can contribute to hypertension and heart disease in adulthood.^6^

Sleep is an important physiological process and plays an essential role in growth, maturation and health during childhood and adolescence.^7^ Paciência et al.^8^ highlight that in adolescence, sleep patterns change physiologically. In their study, the average sleep duration for teenagers ranges from nine hours a day at age 13 to 8.25 hours a day at age 17.^8^ However, in addition to physiological factors, social issues, such as the fast pace of modern life, and behavioral issues, such as the use of technology mainly at night, have contributed to reducing the average duration of sleep, especially among teenagers.^9^ ^,^ ^10^ ^,^ ^11^ On average, teenagers sleep less than 8 hours a night,^10^ ^,^ ^11^ as published in the multicenter study with teenagers entitled Healthy Lifestyle in Europe by Nutrition in Adolescence Study (HELENA), in which 33% of participants aged 12 to 17 years old reported sleeping <8 hours a day.^10^

Many studies already show that the sleep duration variable is an important risk factor for the development of hypertension and other cardiometabolic disorders in children, adolescents and adults.^9^ ^,^ ^12^ ^,^ ^13^ Several biological mechanisms are suggested to be causal in the relationship between sleep duration and elevated blood pressure. Shorter amounts of sleep make the sympathetic nervous system run on high. This hyperactivity of central nervous system functions (hypervigilance) has an effect on the acute increase in sympathetic activity, on the activation of the hypothalamic-pituitary-adrenal axis, and on the renin-angiotensin-aldosterone system, which results in increased blood pressure.^14^ ^,^ ^15^ ^,^ ^16^ ^,^ ^17^ In addition, inadequate sleep can cause an imbalance in circadian rhythms,^18^ as well as a decrease in melatonin production,^19^ which affects blood pressure levels. Most of this research was carried out on samples of adults. Therefore, it is not clear whether these variables are also predictive of high blood pressure in adolescents.

The fact is that the association between sleep duration and blood pressure in adolescence remains uncertain. While some scholars found that short sleep duration was associated with higher blood pressure,^20^ ^,^ ^21^ other studies report a positive association, in which longer sleep duration is related to higher blood pressure levels.^8^ ^,^ ^22^ Furthermore, some do not show any connection at all.^23^ In Brazil, a study was carried out, but its findings were not sufficient to clarify the reality about the relationship between sleep duration and blood pressure in Brazilian adolescents.^24^ Thus, this review aimed to gather and discuss the main epidemiological evidence of the association between sleep duration and blood pressure in adolescents reported in the scientific literature.

## METHOD

This is a systematic review. The search for the studies was carried out in the following databases: Online Medical Literature Search and Analysis System (MEDLINE, via PubMed), Scopus, Web of Science, ScienceDirect and Latin American and Caribbean Literature in Health Sciences (Lilacs). The Virtual Health Library (VHL), the Virtual Adolescent Health Library (Adolec) and the Scientific Electronic Library Online (SciELO) were also consulted.

The search strategy used in the databases included terms selected based on the Health Sciences Descriptors (DeCS) and the Medical Subject Headings (Mesh). The terms were organized into three groups:

- “Sleep duration”, “sleep”.
- “Blood pressure”, “arterial pressure”, “arterial blood pressure”, “hypertension”, “prehypertension”, “high blood pressure”, “elevated blood pressure”.
- “Adolescent”, “teen”, “teenager”, “juvenile”, “youth”, “young people”.

Within each group, the Boolean operator “OR” was used between each term; and between groups, the Boolean operator “AND” was used. The searches took place from July to September 2018.

Studies that met the following criteria were considered eligible: an original article; articles with a cross-sectional or longitudinal design; articles considering adolescents to be individuals aged between ten and 19 years old; articles that presented measures outlining an association between sleep duration (exposure) and blood pressure (outcome); articles that defined blood pressure as prehypertension or high blood pressure, when systolic blood pressure (SBP) or diastolic blood pressure (DBP) was above the 90th percentile and hypertension when SBP or DBP was above the 95th percentile; articles that reported their method of measuring sleep duration and blood pressure; articles published in English, Portuguese or Spanish. The included studies had no restrictions on the sample size or the date of publication. The reference lists of the selected publications were checked for additional publications.

Studies that evaluated adolescents who were pregnant or in specific health conditions (diabetics, chronic kidney patients, those with obstructive sleep apnea or other cardiovascular or sleep-related disorders) were excluded.

According to the eligibility criteria, the studies included in the review were selected and evaluated by the authors. Disagreements were resolved by consensus. From each study, information was extracted and analyzed, such as: author’s name, year of publication, country, study design, sample size, age of participants, sleep duration data collection method, how the sleep duration variable was assessed, blood pressure data collection method, adjustment variables and main results based on the measures of the association between sleep duration and blood pressure variables.

The studies were assessed for methodological quality using the instrument provided by the Agency for Healthcare Research and Quality (AHRQ), which is applicable to cross-sectional studies.^25^ This instrument consists of 11 items, for which the score was zero to 11, eleven being the maximum score. As such, the evaluation of each study was defined as:

- Low quality: zero to three.
- Moderate quality: four to seven.
- High quality: eight to 11.

## RESULTS

**Figura 1:**
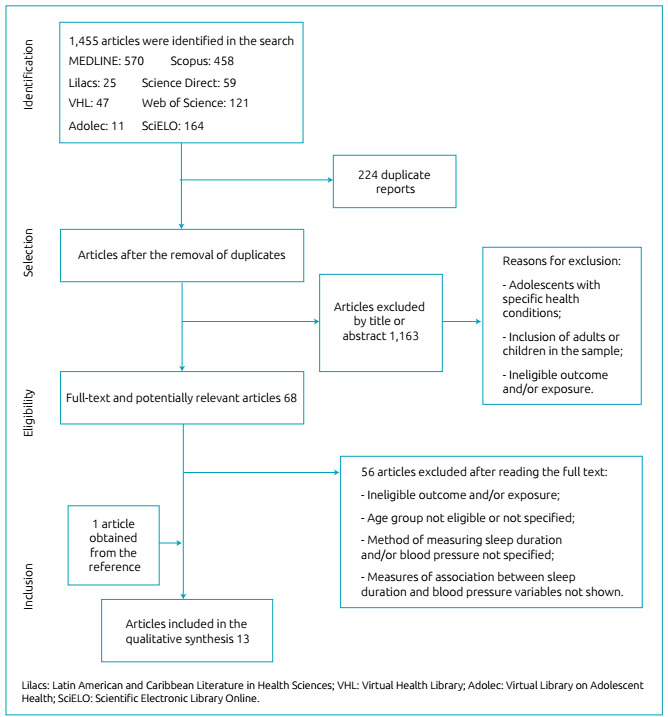
Diagrama de fluxo do processo de seleção dos artigos.

Initially, 1,455 studies were found. A total of 224 articles were excluded, due to duplicity, and 1,163 were excluded because they did not meet the eligibility criteria for screening the title and abstract. A total of 68 potentially relevant articles for full reading were pre-selected. Based on their complete reading, 56 were excluded because they had an ineligible outcome and/or exposure, an ineligible or unspecified age group, they did not present measures that outlined any association between the variables sleep duration and blood pressure, or they did not report the method of measuring sleep duration and/or blood pressure. As such, 12 articles were selected. Then, one article that was identified by reading the references of the selected articles, was added. Thus, at the end of the selection, 13 studies were included in this review, which met the objective and the proposed criteria, as shown in the flow diagram (Figure 1).

Twelve studies had a cross-sectional design,^20^ ^,^ ^21^ ^,^ ^22^ ^,^ ^23^ ^,^ ^24^ ^,^ ^26^ ^,^ ^27^ ^,^ ^28^ ^,^ ^29^ ^,^ ^30^ ^,^ ^31^ while two had a longitudinal design.^8^ ^,^ ^32^ The sample size of the studies varied from 143^26^ to 6,940^20^ individuals. Adolescents from countries on different continents were studied: four studies were conducted in North America (United States),^21^ ^,^ ^27^ ^,^ ^30^ ^,^ ^32^ three were applied in Europe,^8^ ^,^ ^20^ ^,^ ^22^ five were in Asia ^23^ ^,^ ^26^ ^,^ ^28^ ^,^ ^29^ ^,^ ^31^ and only one was in South America (Brazil).^24^ Regarding the evaluation of the quality of the article, three (23%) studies were classified as high quality,^20^ ^,^ ^22^ ^,^ ^29^ around 70% (n=9) of the included studies were considered to be of moderate quality,^8^ ^,^ ^21^ ^,^ ^24^ ^,^ ^26^ ^,^ ^27^ ^,^ ^28^ ^,^ ^29^ ^,^ ^31^ ^,^ ^32^ while only one study (8%) was of low quality.^23^ The main methodological issues that contributed to a moderate or low score involved: the lack of information about how the evaluators of the participating subjects were trained, the percentage of subjects with missing data and how these data were treated, among other aspects regarding the internal validity of the data, as suggested by the AHRQ instrument used.^25^ The characteristics, the main results, according to the evidence of association found, and the methodological quality score of the 13 studies analyzed in this review are described in Tables 1, 2 and 3.

**Table 1:** Characterization and methodological quality score of the studies with evidence of the association between short sleep duration and high blood pressure.

Author (year)	Design Location	Sample Age group	Measurement method	Sleep duration	Adjustment variables	Association between sleep duration and BP in adolescents	Score*
Kuciene et al.^20^ (2014)	Cross-sectional Lithuania	n=6,940 12-15 years old	**Sleep:** self-reported **BP:** oscillometric	<7 h 7-8 h ≥8 h	Age, sex, BMI, physical activity and smoking	Sleep duration <7 h and hypertension: aOR=2.28; sleep duration 7-8 h and hypertension: ORa=1.99; both sexes; for all p <0.001.	9
Javaheri et al.^21^ (2008)	Cross-sectional United States	(n=238) 13-16 years old	**Sleep:** PSG and actigraphy **BP:** PSG and auscultatory	≤6.5 h > 6.5 h	Age, sex, race, BMI, preterm status, and socioeconomic status	Sleep duration <6.5 h and pre-hypertension: OR=2.79, p=0.0366. Sleep duration and SBP: β = -1.74, p = 0.0012. Unadjusted analyzes	7
Wells et al.^24^ (2008)	Cross-sectional Brazil	n=4,452 10-12 years old	**Sleep:** self-reported **BP:** oscillometric	≤8, 8-10, 9, ≥11 h	Maternal education, sex, alcohol, birth weight, smoking during pregnancy, physical activity, socioeconomic status, maternal BMI	Sleep duration and SBP: β =0.31, p=0.03.	5
Au et al.^26^ (2014)	Cross-sectional China	n=143 10-17 years old	**Sleep:** PSG and sleep diary for 7 days **BP:** 24 hours	≤7, 7.01-8, 8.01-9, 9.0-10,> 10 h	Age, sex, BMI, hypertensive parents, hypopnea index (index <5)	Sleep diary: sleep duration and SBP (β = -2.0, p<0.001) and DBP (β = -1.1, p <0.02). PSG: sleep duration and SBP (β =-1.6, p<0.07)	6
Meininger et al.^27^ (2014)	Cross-sectional United States	n=366 11-16 years old	**Sleep:** actigraphy **BP:** 24 hours	Continuous in hours	Age, sex, race, mother’s education, sexual maturation, physical activity, BMI, position during BP measurement	Duration of nighttime sleep and SBP: β =-0.57, p<0.0001. Duration of daytime sleep and SBP: β =-0.73, p <0.001 e PAD β=-0.50, p<0.001	6
Lee and Park^29^ (2014)	Cross-sectional South Korea	n=1,187 12-18 years old	**Sleep:** self-reported **BP:** auscultatory	≤5, 6-7, 8-9, ≥10 h	Age, sex, family, income, caloric intake and physical activity	Sleep duration ≤5 h and high BP: aOR = 2.11 (95%CI 1.22-3.65)	8
Mezick et al.^30^ (2012)	Cross-sectional United States	n=246 14-19 years old	**Sleep:** actigraphy **BP:** 24 hours	Continuous in hours	Age, sex, race and BMI	Sleep duration and SBP and DBP: (both β=-0.17 p=0.01). 1 h increase in sleep duration, 24 h prehypertension and elevated daytime BP (aOR = 0.66, 95%CI 0.46-0.97; aOR=0.65, 95%CI 0.42-0.98), respectively	6
Guo et al.^31^ (2011)	Cross-sectional China	n=4,902 5-18 years old	**Sleep:** reported by parents. **BP:** auscultatory	11-14 years old: <9, 9-10, ≥ 10 h	Age, BMI, physical activity and waist circumference	Sleep duration <9 h and hypertension (boys 11-14 years old): aOR=1.5, p <0.05. Sleep duration and BP (boys 11-14 years old), SBP β = -1.04, p = 0.001, DBP β = -0.55, p=0.030	7
Peach et al.^32^ (2015)	Longitudinal United States	n=541 10-13 years old	**Sleep:** self-reported **BP:** auscultatory	Continuous in hours	Age, sex, race, income, physical activity, eating habits, sexual maturation, attention or behavior problems, depression	Sleep duration on weekdays and weekends and hypertension in boys: β = -0.13, β = -0.05, respectively, for both p <0.01	6

*Methodological quality score of the studies: low quality=0 to 3; moderate quality= 4 to 7 and high quality= 8 to 11; PSG: polysomnography; BMI: body mass index; aOR: adjusted Odds Ratio; OR: Odds Ratio; β: beta coefficient; 95%CI: 95% confidence interval; SBP: systolic blood pressure; DBP: diastolic blood pressure.

**Table 2:** Characterization and methodological quality score of the studies with evidence of association between short sleep duration and high blood pressure (BP).

Author (year)	Design Location	Sample Age group	Measurement method	Sleep duration	Adjustment variables	Association between sleep duration and BP in adolescents	Score*
Paciência et al.^8^ (2016)	Cross-sectional and longitudinal Portugal	n=1,403 13-17 years old	**Sleep:** self-reported **BP:** auscultatory	≤7 h > 7 h	BMI and physical activity	In the cross-sectional analysis (sleep duration and BP at 17 years old, in girls): sleep duration and SBP: β = 0.730, 95%CI 0.005-1.455)	7
Paciência et al.^22^ (2013)	Cross-sectional Portugal	n=1,771 13 years old	**Sleep:** self-reported **BP:** auscultatory	≤8.5 h 8.5-9.5 h ≥9.5 h	*Girls:* caffeine intake, BMI and depressive symptoms. *Boys:* caffeine intake, playing sports	Sleep duration and elevated BP (>90 h) in girls: sleep duration 8.5-9.5 h: aOR = 1.56 (95%CI 1.07-2.27). Sleep duration ≥9.5 h: aOR =1,83 (95%CI 1.23-2.70)	8
Guo et al.^31^ (2011)	Cross-sectional China	n=4,902 5-18 years old	**Sleep:** reported by parents **BP:** auscultatory	15-18 years old: <8, 8-9, ≥9 h	Age, BMI, physical activity and waist circumference	Sleep duration <8 h and hypertension (girls 15-18 years old): aOR=0.46, 95% CI 0.23-0.94, p<0.05	7

BMI: body mass index; SBP: systolic blood pressure; β: beta coefficient; 95%CI: 95% confidence interval; aOR: adjusted Odds Ratio; *methodological quality score of the studies: low quality=0 to 3; moderate quality=4 to 7 and high quality=8 to 11.

**Table 3:** Characterization and score of the methodological quality of the studies with evidence of association between short sleep duration and high blood pressure (BP).

Author (year)	Local Design	Sample Age group	Measurement method	Sleep duration	Adjustment variables	Association between sleep duration and PA in adolescents	Score*
Shaikh et al.^23^ (2010)	Cross-sectional India	n=489 16-19 years old	**Sleep:** self-reported **BP:** oscillometric	≤7 h > 7 h	None	There was no association between sleep duration and BP	3
Azadbakht et al.^28^ (2013)	Cross-sectional Iran	n=5,528 10-18 years old	**Sleep:** reported by parents **BP:** auscultatory	<5 h 5-8 h >8 h	Sex, age, socio-economic status, parents’ education, BMI, family history of chronic diseases, and sedentary lifestyle.	There was no significant association between sleep duration and BP	7

*Methodological quality score of the studies: low quality=0 to 3; moderate quality= 4 to 7 and high quality= 8 to 11.

## DISCUSSION

Evidence about the association between sleep duration and blood pressure in adolescents has been found and discussed in the scientific literature. However, there is still no consensus. There was great diversity in the measurement of the variables, cut-off points, categorization, sample sizes and statistical methods used in the reviewed studies.

The method of measuring sleep duration is important for the robustness of the results, since subjective methods such as self-reporting, parental reporting, questionnaires or sleep diaries can overestimate or underestimate the measure of sleep duration,^9^ ^,^ ^27^ ^,^ ^28^ ^,^ ^33^ while the objective measurement of sleep duration, performed through polysomnography, is considered to be the gold standard.^34^ The polysomnographic study carried out in the laboratory for an entire night is the standard method for monitoring and diagnosing sleep disorders.^35^ In the situation of not having a polysomnography available, actigraphy was used, which is an examination carried out by equipment similar to a clock (actigraph). Through actigraphy, it was possible to estimate the total sleep time, plot a sleep and wake period chart and study the circadian rhythm of an individual who has used the equipment for a specified number of days.^36^ However, research demonstrates sufficient agreement between self-reported measures and objective measures,^18^ ^,^ ^34^ ^,^ ^37^ ^,^ ^38^ suggesting that studies using only questionnaires are also valid.

It was observed that many studies take into account the role of potential confounding factors or effect modifiers in the results, through stratified analyzes in subgroups by age group and/or sex, or adjusting them for several covariates, as has been suggested by other researchers.^18^ ^,^ ^24^ ^,^ ^32^ ^,^ ^39^ However, the study by Shaikh et al.^23^ performed unadjusted analyzes, limiting associations.

This review gathered results from studies that mostly inferred the hypothesis that short sleep duration is significantly associated with the risk of high blood pressure among adolescents.^20^ ^,^ ^21^ ^,^ ^24^ ^,^ ^26^ ^,^ ^27^ ^,^ ^29^ ^,^ ^30^ ^,^ ^31^ ^,^ ^32^ The analysis by Javaheri et al.^21^ revealed that, after adjusting for sex, body mass index (BMI) and socioeconomic status, short sleep duration increased the chance of prehypertension by 2.5 times. In the study by Au et al.,^26^ sleep duration was inversely associated with blood pressure, and the mean reduction of 1 hour in sleep duration was associated with an increase of 2 mmHg in SBP and 1 mmHg in DBP.

The evidence that sleep duration is inversely associated with blood pressure is consistent with recent research results. Quan et al. consider short sleep duration as an important behavioral factor that affects blood pressure in children and adolescents.^40^ In the meta-analysis of Jiang et al.,^41^ the Odds Ratio (OR) of the grouped data indicated that short sleep duration was associated with the risk of high blood pressure (OR=1.51; 95% confidence interval [95% CI] 1.04-2.19, random effects model), mainly in male adolescents (OR = 1.55; 95%CI 1.24-1.93, random effects model).^41^

The underlying mechanism for the association between short sleep duration and elevated blood pressure is not fully understood, but authors have suggested that short sleep could increase blood pressure by causing a misalignment of one’s biological clock, could increase sympathetic nervous system activity and renal sodium retention, and could stimulate physical and psychosocial stressors.^18^ ^,^ ^42^ In addition, short sleep duration is likely to be associated with emotional changes such as irritability, impatience, pessimism, fatigue and stress,^43^ which would make maintaining a healthy lifestyle more difficult when protecting against hypertension.^18^

Taking into account the results of the reviewed studies, it was observed that the association of elevated blood pressure in adolescents has been found at both ends of the sleep duration distribution, as well as in studies with adults.^9^ ^,^ ^12^ In addition to the association between short periods of sleep, some reviewed studies reported evidence for the association between long sleep duration and increased blood pressure in adolescents,^8^ ^,^ ^22^ ^,^ ^31^ Although, when these studies were analyzed in groups, the combined OR indicated a non-significant association between prolonged sleep and high blood pressure (OR=1.04; 95%CI 0.78-1.38, random effects model).^41^

In the study by Guo et al.,^31^ the subgroup of girls aged 15 to 18 who slept <8 hours was significantly less likely to have hypertension compared to participants who slept 8 to 9 hours (OR=0.46 ;95%CI 0.23-0.94) after adjustment, with short sleep duration being a protective factor for hypertension. However, the author emphasizes that there were few individuals in the 15 to 18 age group among the girls (9.5%), limiting the statistical power of this association.

In the study by Paciência et al.^22^ with 13-year-old adolescents, girls with long sleep duration (≥9.5 hours per day) were at higher risk of having higher systolic blood pressure levels when compared to those who slept around 8.5 hours. After adjusting for BMI and caffeine consumption, the magnitude of the association increased and a dose-response effect was observed. An OR=1.56 (95%CI 1.07-2.27) was found among those who slept between 8.5 and 9.5 hours and an OR=1.83 (95%CI 1.23-2.70) was found among those who slept ≥9.5 hours. Among boys, there was a positive association between sleep duration and high blood pressure, but this association was not significant.

A similar result was found by the same authors in another study, in which it was possible to carry out a cross-sectional and longitudinal analysis,^8^ suggesting that the differences in this set of studies reveal a possible J-shaped curve association, in which the higher blood pressure values were also among those with the longest duration of sleep.^8^

This relationship between long sleep durations and elevated blood pressure has not been investigated in depth and is not yet clear in the literature. No biological mechanism has been identified to explain the association between long sleep duration and adverse health outcomes,^12^ however the associations can be explained by confounding factors associated with long sleep duration and/or elevated blood pressure,^39^ ^,^ ^44^ ^,^ ^45^ ^,^ ^46^ ^,^ ^47^ ^,^ ^48^ ^,^ ^49^ indicating that long periods of sleep may be a marker or consequence of poor health rather than a causative risk factor.^39^ ^,^ ^41^

Some studies found no evidence of an association between sleep duration and blood pressure.^23^ ^,^ ^28^ The blood pressure values of Indian adolescents with inadequate sleep duration did not show any significant difference when compared to those with adequate sleep duration.^23^ According to the author, this may have occurred due to the fact that adolescents had an equal amount of physical activity, regardless of the duration of sleep, and that, due to their involvement in physical activity, adolescents deprived of sleep may be maintaining their blood pressure levels similar to those of adolescents who sleep adequately.^23^ The methodological limitations and the statistical method used may have compromised the consistency of its findings, given the low score obtained in the quality assessment described in the results.

The evidence for the association between sleep duration and blood pressure mentioned in this review has limitations due to the characteristics of the studies analyzed. As for the design, there were few longitudinal investigations, which would allow for more robust conclusions. Most of the included studies were cross-sectional. In them, causality can be questioned, as they do not establish the causality of an observed association or temporal order between the variables,^50^ thus enabling a potential bidirectional relationship between the theorized independent and dependent variables, since it has been reported that adolescents with hypertension may have difficulty initiating sleep.^51^

The studies were of different regions and sample sizes, and their respective results could not be extrapolated to a general context, but rather only to the reality of the region in which each research study was conducted. Different methods were used to measure and classify sleep duration and blood pressure. The cutoff points for defining the short or long duration of sleep varied between studies. In some, the variables blood pressure and/or sleep duration were analyzed continuously, and in others they were analyzed categorically. In addition, regression models were adjusted for different variables, which probably also contributed to the inconsistency of the results observed between the studies.

The use of different methods among the revised manuscripts was the reason for a qualitative analysis. This fact, however, did not mean low quality, given that 92% of the studies obtained a moderate or high quality score. In general, the authors of the reviewed studies were judicious in the planning, collection and analysis of data and in the writing of said research, which gave them consistency and quality according to the criteria established by the AHRQ.^25^

Even with the mentioned limitations, it is important to study this issue more deeply and interpret the findings in light of biological plausibility, which brings important evidence that inadequate amounts of sleep may compromise the blood pressure of adolescents. It is worth mentioning that high blood pressure in adolescence is a growing public health problem, as well as a crucial factor in the development of hypertension and other cardiovascular diseases in adulthood.

In conclusion, the main evidence demonstrated by most of the reviewed studies is that short sleep duration is associated with high blood pressure in adolescence. However, there is the possibility of high blood pressure being associated with long sleep duration, but this is not clear in the literature.
